# MCP-1-deficient mice show reduced neuroinflammatory responses and increased peripheral inflammatory responses to peripheral endotoxin insult

**DOI:** 10.1186/1742-2094-5-35

**Published:** 2008-08-15

**Authors:** Wendy L Thompson, William J Karpus, Linda J Van Eldik

**Affiliations:** 1 Department of Cell and Molecular Biology, Northwestern University, Chicago, IL 60611, USA; 2 Department of Pathology, Northwestern University, Chicago, IL 60611, USA; 3 Center for Drug Discovery and Chemical Biology, Northwestern University, Chicago, IL 60611, USA

## Abstract

**Background:**

An endotoxin insult mimics a severe peripheral infection and recent evidence suggests that a single exposure can cause long-term cognitive deficits. A peripheral injection of LPS results in production of pro-inflammatory cytokines, such as IL-1β and TNF-α, in the brain and periphery and these cytokines mediate many effects of the acute phase response including activation of the HPA axis. The chemokine MCP-1 is highly expressed during endotoxemia and although much is known about the importance of MCP-1 in peripheral inflammatory responses to LPS, information about MCP-1 and CNS responses to peripheral LPS is lacking.

**Methods:**

C57Bl/6 mice were administered LPS by intraperitoneal (i.p.) injection, serum and brains were collected at several time points, and the time course of MCP-1 protein up-regulation was measured. To examine the role of MCP-1 in activation of the brain during acute systemic inflammation, we injected MCP-1 knockout (MCP-1^-/-^) or control C57Bl/6 (MCP-1^+/+^) mice with LPS i.p. and measured the levels of selected cytokines and chemokines in serum and brain extracts 6 hours later. Activated microglia were examined by CD45 immunohistochemistry, and serum corticosterone and ACTH levels were measured by enzyme immunoassay.

**Results:**

We report that LPS injection induces a robust increase in MCP-1 protein levels in serum and brain, with peak brain levels reached at 6 hrs after LPS administration. MCP-1^-/- ^mice injected with LPS showed higher levels of serum IL-1β and TNF-α compared to LPS-treated MCP-1^+/+ ^mice. In contrast, these MCP-1^-/- ^mice showed significantly lower inductions of brain pro-inflammatory cytokines and chemokines, fewer activated microglia, and a reduction in serum corticosterone levels.

**Conclusion:**

MCP-1^-/- ^mice have decreased brain inflammation after a peripheral LPS insult, despite an exaggerated peripheral response. These data demonstrate an important role for MCP-1 in regulation of brain inflammation after peripheral endotoxemia.

## Background

The mammalian body responds rapidly to an infection from invading pathogens by activation of the innate immune system. Bi-directional communication between the immune system and the brain is necessary for activation of the acute phase response and efficient clearance of the invading organism. Through incompletely understood mechanisms and pathways, the cells of the brain become activated and turn on specific neural pathways that regulate the acute phase response whose manifestations include fever, sickness behaviors, and activation of the hypothalamic-pituitary-adrenal (HPA) axis. Fever and sickness behaviors such as lethargy, anorexia, anhedonia, and social isolation together represent evolutionary adaptations to help the organism fight the invading pathogen [1,2]. Likewise, activation of the HPA axis and subsequent production of glucocorticoids are critical to the organism's survival by regulating the body's immune system responses [3-5].

The endotoxin lipopolysaccharide (LPS) is found in the outer cell wall of Gram-negative bacteria and when injected systemically can generate many features of the acute phase response [2,4] and has therefore been used extensively as a model for peripherally induced inflammation. Systemic LPS causes an increase in production of pro-inflammatory cytokines, such as interleukin (IL)-1β, tumor necrosis factor (TNF)-α, and IL-6, in the periphery by immune cells such as monocytes and tissue macrophages [6]. These peripherally produced cytokines may transfer an inflammatory signal to the brain in several ways: activation in areas of the brain with a leaky blood brain barrier (BBB), direct transport of cytokines across the BBB, or a neural route [6]. Certain cells of the brain express the receptor for LPS, toll like receptor 4 [7], and these cells may be directly activated by LPS. Further, migration of blood leukocytes into the central nervous system (CNS) [8] may also contribute to brain activation. These peripheral inflammatory signals stimulate certain brain cells to endogenously express the same pro-inflammatory cytokines [2,9-11]. Evidence that innate immune cells of the brain can be activated by these cytokines is demonstrated by the observation that a single injection of IL-1β or TNF-α into selected regions of the brain results in sickness behaviors or activation of the HPA axis, and receptors for these cytokines are expressed in several brain regions [2,12].

A number of chemotactic cytokines or chemokines are also upregulated in the periphery and brain after a peripheral injection of LPS [13-17]. Chemokines are essential in cell recruitment and trafficking during inflammation, are involved in regulating leukocyte movement into the CNS during pathology, and may also affect the BBB permeability [18-20]. The chemokine monocyte chemoattractant protein-1 (MCP-1; also called CCL2) plays an essential role in several peripheral and CNS inflammatory disorders characterized by mononuclear cell infiltrates, and inhibiting MCP-1 in mouse models of atherosclerosis, stroke and experimental autoimmune encephalomyelitis resulted in a decrease in inflammatory cell recruitment and reduced disease severity [21-23]. Similarly, there is decreased myeloperoxidase activity and attenuated liver and lung injury when MCP-1 is inhibited after cecal ligation and puncture or after systemic LPS challenge [24,25]. However, prior studies [15,26-28] have also shown that inhibition of MCP-1 activity using neutralizing antibodies or in knock-out mice lacking MCP-1 resulted in a dysregulation of the cytokine balance and increased mortality in models of sepsis induced by cecal ligation and puncture or LPS, and *Salmonella *infection. Additional evidence for a protective or reparative role of MCP-1 includes the observations that pre-treatment of mice with recombinant MCP-1 led to a reduction in mortality and pro-inflammatory cytokine induction after a high dose of LPS [15], and that treatment of an astrocyte cell line with MCP-1 led to the induction of the neurotrophic factor, basic fibroblast growth factor [29]. Thus, MCP-1 may play several vital roles besides one in cell recruitment and may have either a protective or detrimental role depending on the inflammatory stimulus, cell type, or disease state.

Although much is known about the importance of MCP-1 in peripheral inflammatory responses to LPS, there is little information about MCP-1 and CNS responses to peripheral LPS. The aim of this study, therefore, was to examine the role of the chemokine MCP-1 in activation of the brain during acute systemic inflammation. To do this, we examined the effects of inhibiting MCP-1 on brain inflammation by using a MCP-1 knock-out (MCP-1^-/-^) mouse [30]. Interestingly, we discovered that although the cytokine response was significantly greater in the serum of LPS-treated MCP-1^-/- ^mice compared to LPS-treated wild-type MCP-1^+/+ ^mice, there was a hyporesponse of the immune cells in the brain of the knockout mice. Specifically, in the hippocampus and cortex of the LPS-injected MCP-1^-/- ^mice, there was a decrease in microglia activation as measured by CD45 immunoreactivity and a decrease in cytokine and chemokine levels. This decrease in microglia activation was accompanied by a reduction in serum corticosterone levels. These data suggest that MCP-1 plays a critical role either directly or indirectly in activating the brain during systemic endotoxemia.

## Methods

### Animals

Female C57Bl/6 (MCP-1^+/+^) mice were purchased from Harlan (Indianapolis, IN). MCP-1^-/- ^mice were kindly provided by Dr. Barrett J. Rollins (Dana Farber Cancer Institute, Boston, MA) and have been backcrossed eight times on the C57Bl/6 background [30]. All mice were maintained in a pathogen-free facility (the Center for Comparative Medicine) at Northwestern University, and had access *ad libitum *to standard laboratory chow and water. All animal procedures were approved by the Northwestern Animal Care and Use Committee.

### MCP-1 time course experiments

Adult female MCP-1^+/+ ^mice (20–25 g) were injected intraperitoneally (i.p.) with 5 mg/kg LPS (*Salmonella typhimurium*; Sigma, St. Louis, MO). Control mice received an equivalent volume of saline i.p. The dose of LPS was chosen for the time course experiments so that MCP-1 measurements could be made at longer time points (18 hr and 24 hr) without any mortality. At various times after LPS, mice were anesthetized with pentobarbital (50 mg/kg) and blood was drawn by intracardiac puncture, allowed to clot and centrifuged for serum preparation. Mice were perfused with a HEPES buffer (10 mM, pH 7.2) containing a protease inhibitor mixture (1 μg/ml leupeptin, 100 μM sodium orthovanadate, 1 mM phenylmethylsulphonylfluoride, 1 μg/ml pepstatin, 1 μg/ml aprotinin), brains were removed and hippocampus, entorhinal cortex, and frontal cortex were dissected out. Brain homogenates were prepared by Dounce homogenization in the HEPES buffer containing 0.1% Triton-X 100 and the protease inhibitor mixture. Homogenates were sonicated for 10 seconds on ice, centrifuged at 10,000 × g for 20 minutes, supernatants were collected, and protein levels were determined using the BCA protein assay reagent kit (Pierce, Milwaukee, WI). Serum levels of MCP-1 were measured using a duo-set ELISA (R&D Systems, Minneapolis, MN) per the manufacturer's instructions, and MCP-1 levels in brain supernatants were measured using a multiplex assay per the manufacturer's instructions (Meso-Scale Discovery (MSD), Gaithersburg, MD).

### MCP-1^-/- ^and MCP-1^+/+ ^experiments

Mice were injected i.p. with 10 mg/kg LPS. Control mice received an equivalent volume of saline i.p. At 6 hours after LPS or saline treatment, mice were anesthetized and blood and brains collected and dissected as described above. Serum IL-1β and TNF-α levels were measured using a duo-set ELISA (R&D Systems), and IL-1β and TNF-α levels in brain supernatants were measured using a multiplex assay per the manufacturer's instructions (MSD, Gaithersburg, MD).

### Brain multi-analyte profile

Entorhinal cortex supernatants were analyzed in a multi-analyte profile (Rodent MAP™ Antigens, Version 1.6) by Rules-Based Medicine, Austin, TX. The least detectable dose (LDD) was determined as the mean + 3 standard deviations of 20 blank readings. The LDDs for each of the analytes are: eotaxin (2.5 pg/ml), MCP-3 (6.3 pg/ml), MIP-1α (45 pg/ml), MIP-1β (16 pg/ml), MIP-1γ (15 pg/ml), MIP-3β (93 pg/ml).

### Serum corticosterone and ACTH EIA

Mice were injected with LPS (10 mg/kg) or saline i.p. between 0830 and 1100 h and sacrificed 6 hours later. Levels of serum corticosterone were measured using an EIA kit per the manufacturer's instructions (Assay Designs, Ann Arbor, MI). The assay sensitivity was 27.0 pg/ml. Levels of serum ACTH were measured using an EIA kit per the manufacturer's instructions (Bachem, Torrance, CA). The minimal detectable ACTH concentration was 0.04 ng/ml.

### Histology and immunostaining

After perfusion and dissection, one hemibrain of each mouse was fixed in 4% paraformaldehyde/PBS and cryopreserved in 30% sucrose/PBS containing 0.02% sodium azide. Brains were sectioned coronally on a freezing microtome at 30 μm. Every 24^th ^section was collected in 0.1 M phosphate buffer, pH 7.6 and these free-floating sections were then incubated overnight at room temperature with rat anti-CD45 antibody (1:2000; Serotec). After washes, the sections were incubated with secondary biotinylated goat anti-rat IgG (Jackson ImmunoLabs) at 1:2000 for 2 h at room temperature. The Vector Laboratories (Burlingame, CA) ABC kit was used with DAB as chromogen to visualize the reaction product. The sections were then mounted on charged slides, dehydrated in a series of alcohols, cleared in xylene, and coverslipped. The slides were then coded and 10–15 random pictures were taken by a blinded observer using the Fractionator Probe in the Stereo Investigator 7 program (MBF Bioscience, Williston, VT) from at least 2 sections of hippocampus and cortex from each mouse brain. This approach was used to capture unbiased images throughout the cortex and hippocampus. NIH Image J software was used to measure the intensity of CD45 immunoreactivity in the hippocampus and cortex. The random images were converted to a grayscale 8-bit picture using Image J, and the grayscale image was then converted to a binary mode (black and white) and the mean density for all pixels in the chosen field was calculated. Grayscale values below the cut-off (CD45 immunoreactivity) become black and those above become white. The mean density readout is a number between 0 (pure black) and 255 (pure white), and was subsequently converted to a scale between 0 (pure white) and 100 (pure black). The mean pixel densities of at least 7 pictures per brain region were averaged per mouse.

### Statistical analyses

Experimental and control groups were compared using one-way ANOVA with the Newman-Keuls *post hoc *analysis using a statistical software package (GraphPad Prism, version 4.0; GraphPad Software, San Diego, CA). Statistical significance was assumed when p < 0.05.

## Results

### Time course of MCP-1 protein expression in serum and brain

**Figure 1:**
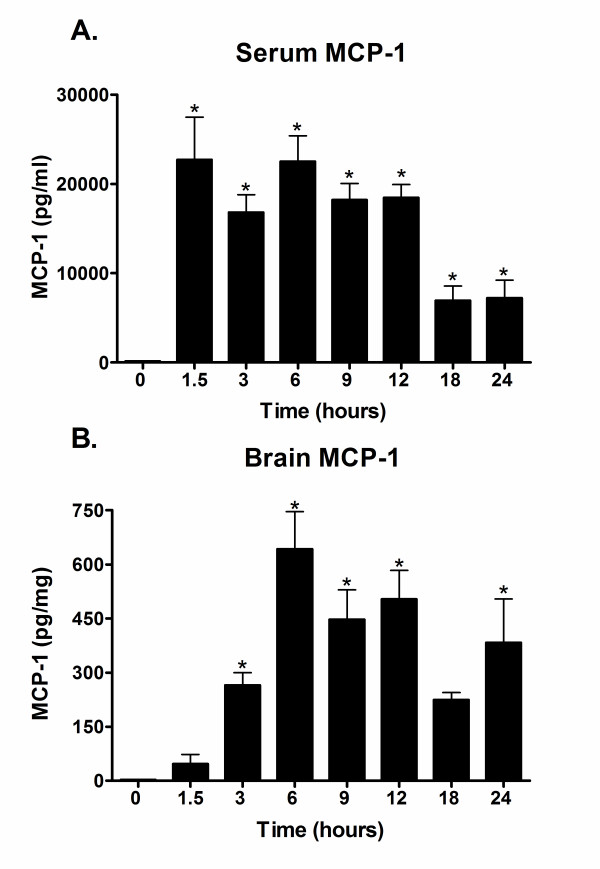
**MCP-1 is increased in a time-dependent manner in serum and brain after LPS treatment**. C57Bl/6 mice were injected i.p. with LPS, and serum and brain frontal cortex were collected at 1.5, 3, 6, 9, 12, 18, and 24 hours after LPS treatment. The 0 hr time point represents saline-injected controls. MCP-1 protein levels were measured in serum (A) and brain (B) as described in Methods. Values are represented as mean ± SEM of duplicate determinations per sample and are pooled from two independent experiments, n = 2–9 mice per time point. * represents significant difference from 0 hr control, p < 0.05.

To define the time course of MCP-1 protein production in response to a peripheral injection of LPS, we measured MCP-1 levels in the serum and in brain frontal cortex at various time points after i.p. administration of LPS to C57Bl/6 mice. The levels of MCP-1 in the serum showed a robust, significant increase by 1.5 hours, and remained significantly elevated over controls through 24 hours (Fig. 1A). In the brain, MCP-1 levels were significantly upregulated by 3 hours, peaked at 6 hours, and slowly waned to a lower but still significantly elevated level at 24 hrs (Fig. 1B). Specifically, by 3 hours, the brain MCP-1 production was at 47% of the maximum level seen at 6 hours, and at 24 hours was still at 61% of the maximum (Fig. 1B). Due to the limited number of MCP-1^-/- ^mice available, in all subsequent experiments we used a single time point of 6 hours. Six hours after LPS treatment represents a point of robust production of MCP-1 protein in the brain and at this time point we were also able to measure significant levels of the cytokines IL-1β and TNF-α in both the brain and serum (data not shown).

### Inhibition of MCP-1 increases peripheral pro-inflammatory cytokine production

**Figure 2:**
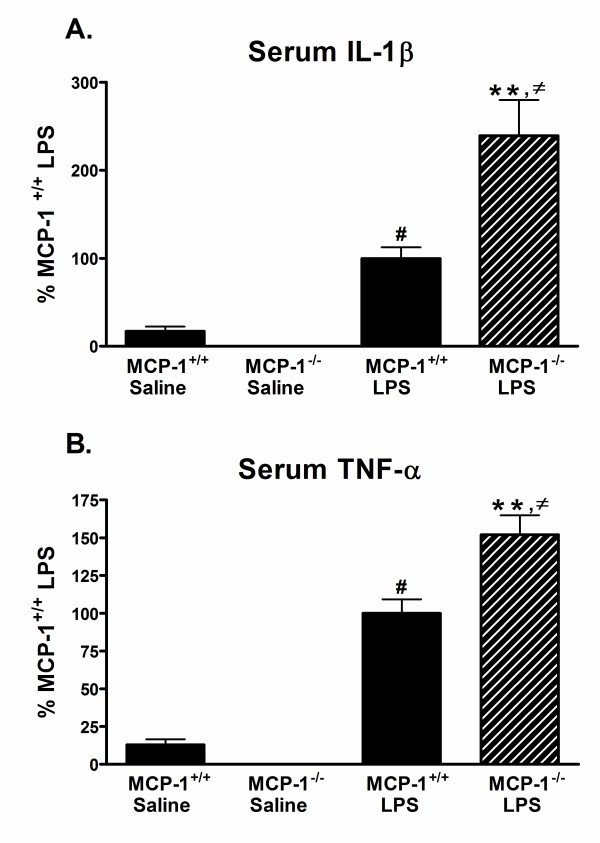
**Pro-inflammatory cytokines IL-1β and TNF-α are increased in serum from MCP-1^-/- ^mice after LPS treatment**. MCP-1^+/+ ^or MCP-1^-/- ^mice were injected i.p. with LPS or saline, and serum was collected 6 hours later. IL-1β (A) and TNF-α (B) levels were measured by ELISA as described in Methods. Values are represented as mean ± SEM of duplicate determinations and are pooled from two independent experiments, n = 5–11 mice per group. LPS-injected MCP-1^+/+ ^mice mean value is 577.6 pg/ml (IL-1β) and 573.4 pg/ml (TNF-α). ** represents significant difference between LPS-treated MCP-1^+/+ ^mice and LPS-treated MCP-1^-/- ^mice, p < 0.001; # represents significant difference between saline-treated MCP-1^+/+ ^mice and LPS-treated MCP-1^+/+ ^mice, p < 0.05; and ≠ represents significant difference between saline-treated MCP-1^-/- ^mice and LPS-treated MCP-1^-/- ^mice, p < 0.001.

Previous studies have demonstrated a critical role for the pro-inflammatory cytokines IL-1β and TNF-α in disease pathogenesis during endotoxemia [31,32]. We performed experiments to determine whether MCP-1 regulates the production of these pro-inflammatory mediators during treatment with LPS. To do this, MCP-1^-/- ^mice or wild-type MCP-1^+/+ ^mice were injected i.p. with LPS or saline, and serum was obtained 6 hours later. As shown in Fig. 2A, treatment with LPS significantly increased IL-1β protein levels above saline controls in both the MCP-1^+/+ ^and MCP-1^-/- ^mice, but the LPS-injected MCP-1^-/- ^mice showed a 2.4 fold increase in IL-1β protein above that seen in the LPS-injected MCP-1^+/+ ^mice. Similar to IL-1β, serum TNF-α was also significantly increased in LPS-injected mice compared to saline controls, with the LPS-injected MCP-1^-/- ^mice showing a 1.5 fold higher increase in TNF-α compared to MCP-1^+/+ ^mice (Fig. 2B). These data confirm prior studies from our lab [27] and others [15] that found an increase in TNF-α in serum of MCP-1 deficient mice challenged with LPS.

### Inhibition of MCP-1 decreases brain inflammation

**Figure 3:**
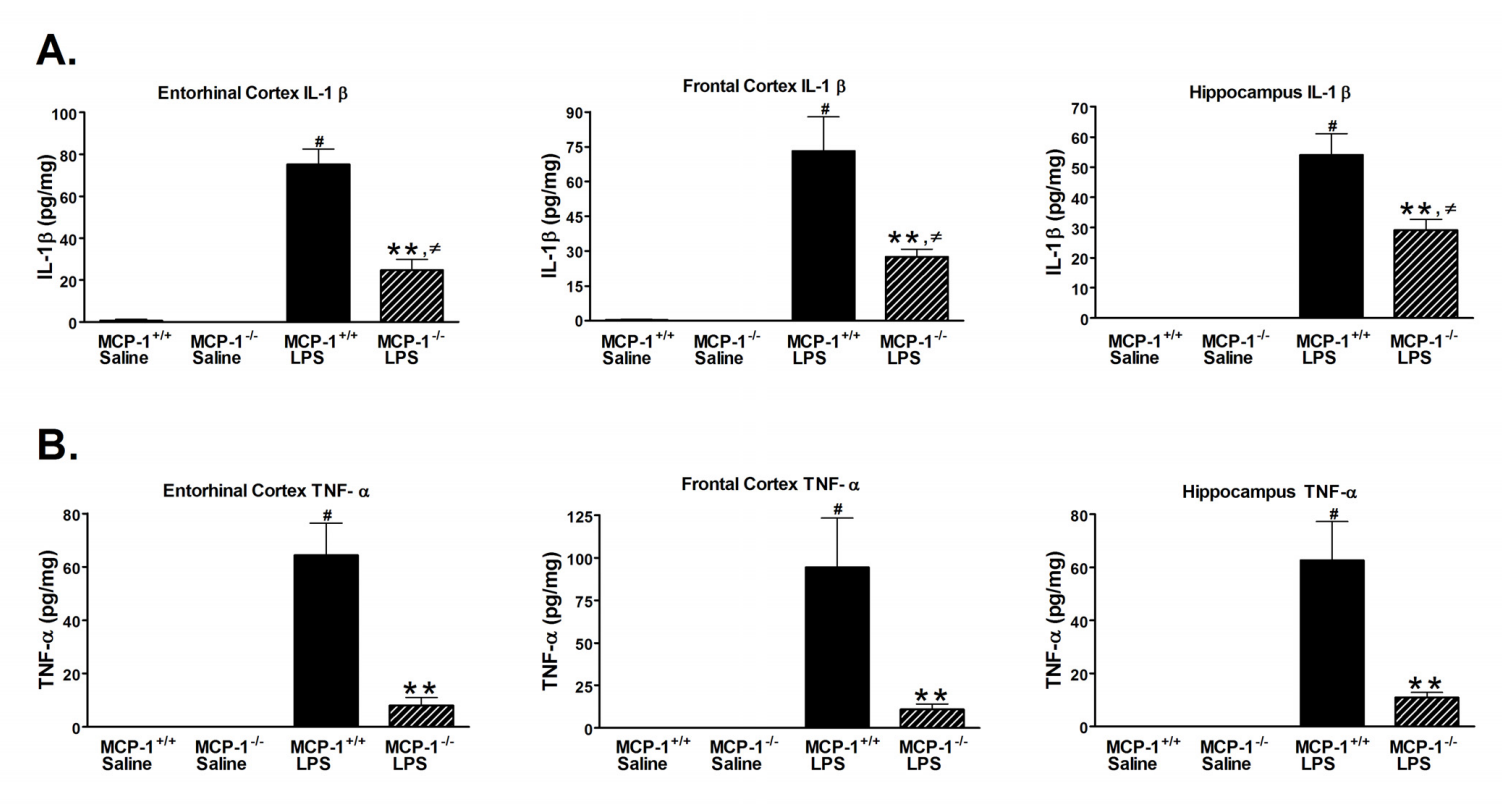
**Pro-inflammatory cytokines IL-1β and TNF-α are decreased in brains of MCP-1^-/- ^mice after LPS treatment**. MCP-1^+/+ ^or MCP-1^-/- ^mice were injected i.p. with LPS or saline, and brains were removed and dissected 6 hours later. IL-1β (A) and TNF-α(B) levels in entorhinal cortex, frontal cortex, and hippocampus supernatants were measured by MSD as described in Methods. Values are represented as mean ± SEM of duplicate determinations and are pooled from two independent experiments, n = 5–11 mice per group. ** represents significant difference between LPS-treated MCP-1^+/+ ^mice and LPS-treated MCP-1^-/- ^mice, p < 0.001; # represents significant difference between saline-treated MCP-1^+/+ ^mice and LPS-treated MCP-1^+/+ ^mice, p < 0.001; and ≠ represents significant difference between saline-treated MCP-1^-/- ^mice and LPS-treated MCP-1^-/- ^mice, p < 0.05.

Stimulation of the brain and neural pathways is essential for activation of the HPA axis, activation of sickness behaviors, and regulation of the peripheral immune system. To examine the role of MCP-1 in activating the brain during endotoxemia, we quantified the amount of brain inflammation by first measuring production of the pro-inflammatory cytokines IL-1β and TNF-α in three brain regions. MCP-1^-/- ^or MCP-1^+/+ ^mice were injected i.p. with LPS or saline, brains were harvested and dissected 6 hours later, and IL-1β and TNF-α levels in entorhinal cortex, frontal cortex, and hippocampal supernatants were measured. These brain regions were selected because the hippocampus and cortex are areas affected by neuroinflammation in chronic neurodegenerative disorders such as Alzheimer's disease. As expected, LPS treatment induced an increase in the levels of IL-1β and TNF-α in all brain regions compared to saline-treated mice (Fig. 3). However, unexpectedly we found that the IL-1β and TNF-α response to LPS was lower in all brain regions in the MCP-1^-/- ^mice compared to the MCP-1^+/+ ^mice (Fig. 3). Specifically, the IL-1β protein levels in the entorhinal cortex, frontal cortex, and hippocampus of the LPS-injected MCP-1^-/- ^mice were 32.9%, 37.7%, and 53.9% of the respective LPS-injected MCP-1^+/+ ^mice (Fig. 3A). There was an even more striking reduction in the TNF-α protein levels in the LPS-injected MCP-1^-/- ^mice, where the entorhinal cortex, frontal cortex, and hippocampus levels were 12.5%, 11.5%, and 17.4% of the respective levels from the LPS-injected MCP-1^+/+ ^mice (Fig. 3B). Similar results for IL-1β and TNF-α were found in the cerebellum (data not shown). The anti-inflammatory cytokine IL-10, although significantly upregulated with LPS treatment, was not changed between the LPS-treated MCP-1^-/- ^mice and LPS-treated MCP-1^+/+ ^mice (data not shown).

**Figure 4:**
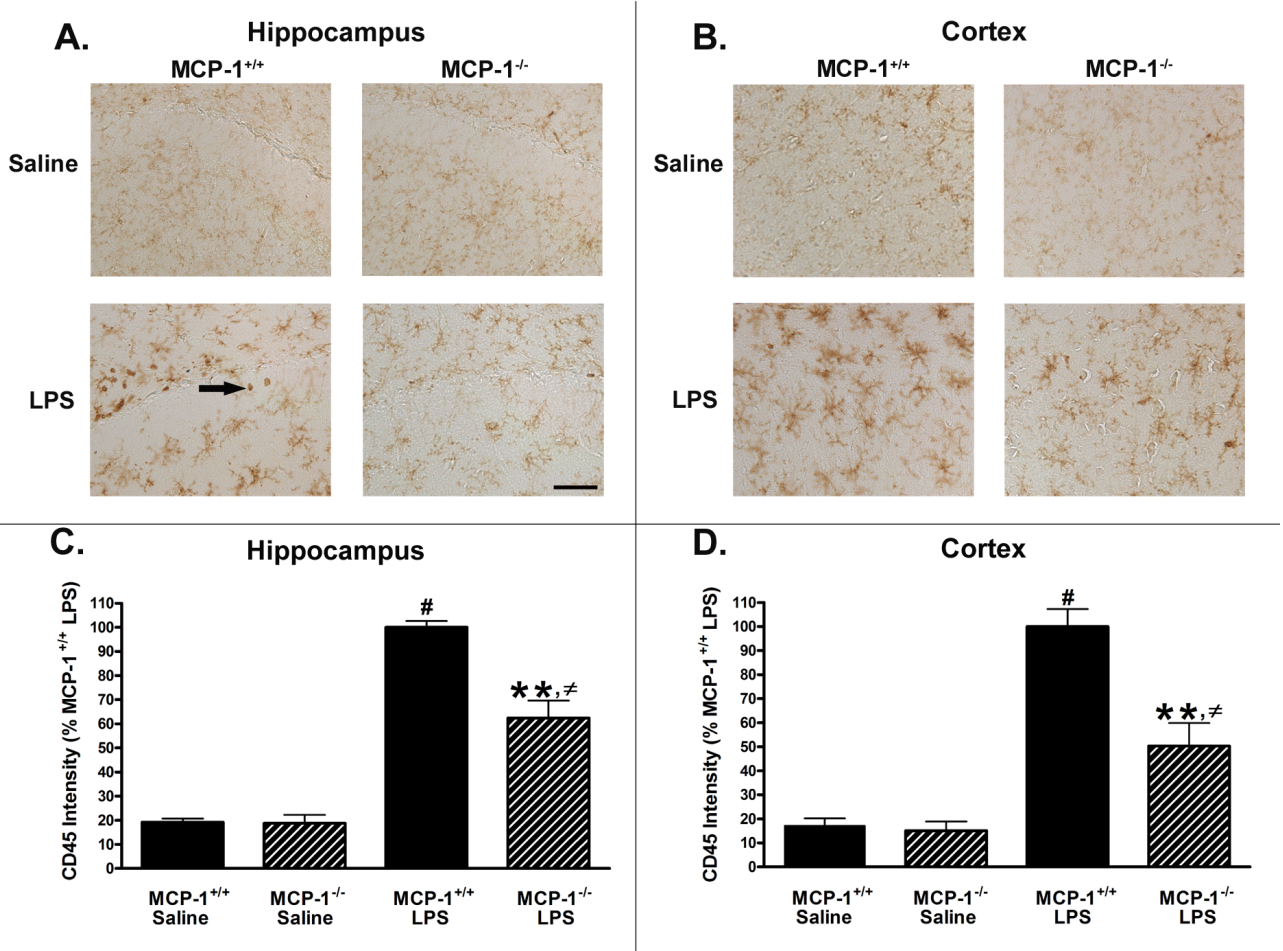
**Brain CD45 immunoreactivity is reduced in MCP-1^-/- ^mice after LPS treatment**. MCP-1^-/- ^and MCP-1^+/+ ^mice were injected i.p. with LPS or saline, and brains were removed 6 hours later. Brain sections (30 μm) were stained with anti-CD45 as described in Methods. Fields of view were randomly generated using StereoInvestigator for 2–3 sections per mouse and photographed at 20× magnification using a Nikon Eclipse 80 i microscope. Arrow shows example of morphologically identified leukocytes. Immunoreactive density was measured on at least 7 pictures from both the hippocampus (A) or cortex (B) per mouse and analyzed by Image J. Values for the hippocampus (C) and cortex (D) are represented as mean ± SEM, n = 5 mice per group. ** represents significant difference between LPS-treated MCP-1^+/+ ^mice and LPS-treated MCP-1^-/- ^mice, p < 0.001; # represents significant difference between saline-treated MCP-1^+/+ ^mice and LPS-treated MCP-1^+/+ ^mice, p < 0.001; and ≠ represents significant difference between saline-treated MCP-1^-/- ^mice and LPS-treated MCP-1^-/- ^mice, p < 0.01. Scale bar = 50 μm.

Microglial cells and perivascular and meningeal macrophages are the main sources of IL-1β in the brain after peripheral LPS injections [2]. Since there was a decrease in IL-1β and TNF-α in the brains of the MCP-1^-/- ^mice, we investigated whether this corresponded to a decrease in activated microglia in these mice. To examine whether there was a decrease in activation of these immune cells, we performed CD45 immunohistochemistry on the hippocampal and cortical sections of saline- or LPS-injected MCP-1^-/- ^or MCP-1^+/+ ^mice. As shown in Fig. 4A, B, microglia in the saline-injected mice appear phenotypically to be in the classic resting state. After LPS injection, the mouse brains show more intense staining and a phenotype consistent with activated microglia: enlarged and de-ramified cell bodies. Quantification of the CD45 immunoreactivity showed that peripheral LPS injection resulted in a significant increase in CD45 immunoreactivity over saline-injected controls at 6 hours (Fig. 4C, D). In addition, there is a significantly lower amount of CD45 immunoreactivity in both the hippocampus and cortex of the LPS-injected MCP-1^-/- ^mice compared to LPS-injected MCP-1^+/+ ^mice (Fig. 4C, D). We also observed fewer stained cells resembling leukocytes in and surrounding the hippocampus of the LPS-injected MCP-1^-/- ^mice (1 of 4 mice) compared to LPS-injected MCP-1^+/+ ^mice (4 of 5 mice) (e.g. see arrow in Fig. 4A for examples). These data suggest that there was a decrease in activation and number of immune cells in the brains of the LPS-injected MCP-1^-/- ^mice compared to LPS-injected MCP-1^+/+ ^mice.

**Figure 5:**
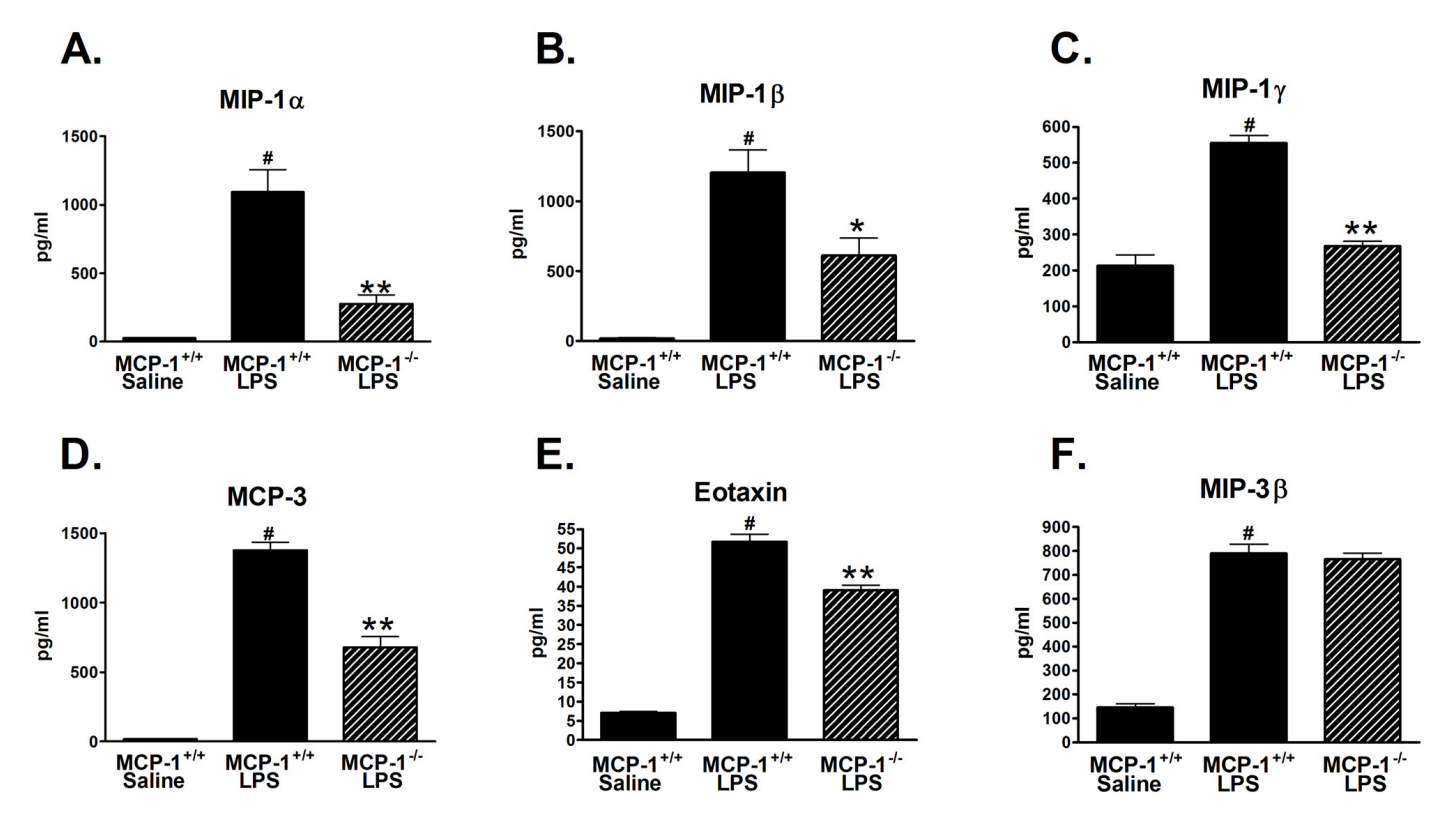
**CC Chemokines are decreased in brains of MCP-1^-/- ^mice after LPS treatment**. MCP-1^-/- ^and MCP-1^+/+ ^mice were injected i.p. with LPS or saline (MCP-1^+/+ ^mice only), and brains were removed and dissected 6 hours later. Entorhinal cortex was homogenized and supernatant assayed by Rules-Based Medicine for MIP-1α (A), MIP-1β (B), MIP-1γ (C), MCP-3 (D), eotaxin (E), and MIP-3β (F). Values are represented as mean ± SEM, n = 4–6 mice per group. ** or * represents significant difference between LPS-treated MCP-1^+/+ ^mice and LPS-treated MCP-1^-/- ^mice, **p < 0.001, *p < 0.01; # represents significant difference between saline-treated MCP-1^+/+ ^mice and LPS-treated MCP-1^+/+ ^mice, p < 0.001.

High doses of peripheral LPS injections cause increases in many pro-inflammatory mediators in the brain, including chemokines. Based on our observations of a decrease in microglia activation in the brains of the LPS-injected MCP-1^-/- ^mice, we examined if other chemokine proteins were also reduced. Because previous data demonstrated that the brain cytokine levels in saline-injected mice were low or below detection limits, we used only saline-injected MCP-1^+/+ ^mice and compared the LPS-induced levels of several chemokines in the MCP-1^-/- ^and MCP-1^+/+ ^mice. As shown in Fig. 5A–E, MIP-1α (CCL3), MIP-1β (CCL4), MIP-1γ (CCL9), MCP-3 (CCL7), and eotaxin (CCL11) proteins were all increased with LPS treatment, but were significantly decreased in the LPS-treated MCP-1^-/- ^mice compared with LPS-treated MCP-1^+/+ ^mice. This reduced response to LPS appeared to be relatively selective to only certain chemokines as there was no difference in MIP-3β (CCL19) protein levels between the LPS-treated MCP-1^-/- ^mice and LPS-treated MCP-1^+/+ ^mice (Fig. 5F). A variety of other analytes measured in the entorhinal cortex supernatants (data not shown) were either increased in the LPS-treated MCP-1^-/- ^mice (IL-3, IL-4, IL-12p70, myeloperoxidase), or not significantly different between the LPS-treated MCP-1^-/- ^mice and the LPS-treated MCP-1^+/+ ^mice (CD40, M-CSF, IL-2, IL-10), further demonstrating selectivity of the response of the MCP-1^-/- ^mice. Altogether, these data show that the inhibition of MCP-1 results in a decrease in several inflammatory cytokines and chemokines in the brain and a decreased activation of microglia after peripheral LPS administration.

### Inhibition of MCP-1 is associated with decreased serum corticosterone levels

**Figure 6:**
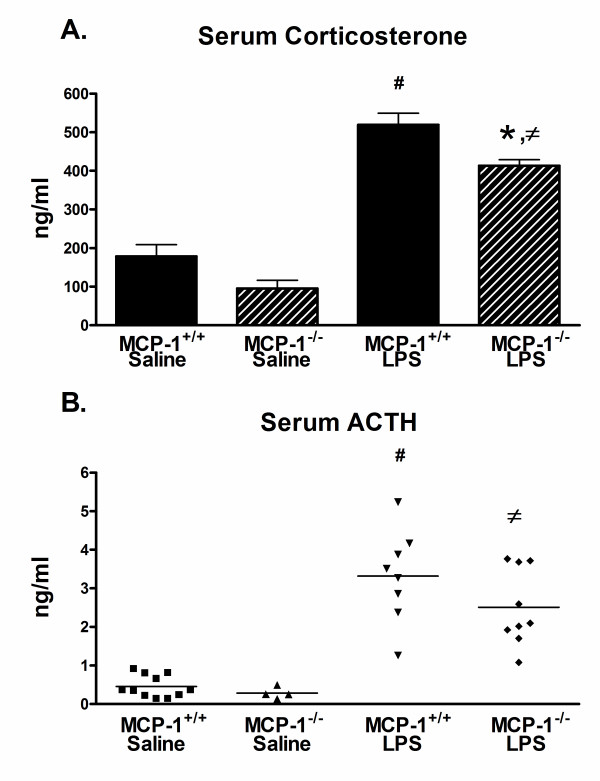
**Serum corticosterone levels are reduced in MCP-1^-/- ^mice after LPS treatment**. MCP-1^-/- ^and MCP-1^+/+ ^mice were injected i.p. with LPS or saline, and serum was collected 6 hours later. Corticosterone (A) or ACTH (B) levels were measured by EIA as described in Methods. Values are represented as mean ± SEM of singlet determinations and are pooled from two independent experiments, n = 4–11 mice per group. * represents significant difference between LPS-treated MCP-1^+/+ ^mice and LPS-treated MCP-1^-/- ^mice, p < 0.01; # represents significant difference between saline-treated MCP-1^+/+ ^mice and LPS-treated MCP-1^+/+ ^mice, p < 0.001; and ≠ represents significant difference between saline-treated MCP-1^-/- ^mice and LPS-treated MCP-1^-/- ^mice, p < 0.001.

In the brain, the pro-inflammatory cytokines IL-1β and TNF-α have been linked to activation of the HPA axis [4,12]. Since we found decreased inflammation and cytokine production in brains of the LPS-treated MCP-1^-/- ^mice, we hypothesized that this hypoactivation might affect production of glucocorticoids, which is a downstream product from activation of the HPA axis. To test this, we collected serum from MCP-1^-/- ^or MCP-1^+/+ ^mice injected with LPS or saline after 6 hours, and measured levels of corticosterone, the primary glucocorticoid present in mice [12]. As shown in Fig. 6A, corticosterone levels were increased after LPS treatment, but were significantly lower in the LPS-injected MCP-1^-/- ^mice compared to the LPS-injected MCP-1^+/+ ^mice. Corticosterone levels in the saline-injected MCP-1^-/- ^mice were also somewhat lower than the saline-injected MCP-1^+/+ ^mice, but this difference was not significantly different. A decreased production of corticosterone in response to LPS may be due to a primary adrenal insufficiency affecting the adrenal glands directly or a secondary adrenal insufficiency which may be caused by a decrease in ACTH, a hormone produced by the pituitary gland and largely responsible for stimulating corticosterone [33]. To test this we measured serum ACTH levels and found that there was a significant increase after LPS treatment (Fig. 6B). There was also a decrease in average serum ACTH levels in the LPS-injected MCP-1^-/- ^mice compared to LPS-injected MCP-1^+/+ ^mice, although this did not reach significance due to high inter-individual variability (Fig. 6B). These data suggest that MCP-1 insufficiency in LPS-treated mice results in reduced corticosterone production.

## Discussion

We report here that MCP-1^-/- ^mice show a decreased upregulation of brain cytokine and chemokine production and decreased activation of brain immune cells after systemic LPS treatment compared to wild-type MCP-1^+/+ ^mice. These decreased responses were correlated with lower serum corticosterone levels in the knockout mice. In contrast, there were significantly higher levels of the pro-inflammatory cytokines IL-1β and TNF-α in the serum of LPS-injected MCP-1^-/- ^mice compared to LPS-injected MCP-1^+/+ ^mice. These data suggest that during endotoxemia MCP-1 is necessary for full activation of the brain immune cells and production of endogenous brain inflammatory mediators. Further, the lower levels of corticosterone, a potent immunosuppressant, in the serum of MCP-1^-/- ^mice may be one factor involved in the increased immune response in the periphery in these mice.

The mechanistic relationship between MCP-1 and the production of peripheral inflammatory mediators is not completely understood. Previous studies have shown that MCP-1 knockout mice or wild type mice treated with MCP-1 neutralizing antibodies show a reduction in the serum levels of the anti-inflammatory cytokine IL-10 and increased levels of the pro-inflammatory cytokine TNF-α after LPS injection [15,27]. Further, when recombinant mouse MCP-1 is administered to mice receiving LPS, IL-10 levels are increased and TNF-α levels are decreased in the serum [15]. Our data showing increased levels of IL-1β and TNF-α in the serum of LPS-injected MCP-1^-/- ^mice compared to wild-type mice are consistent with these previous studies, and suggest that MCP-1 may be involved in controlling the balance between production of pro-inflammatory and anti-inflammatory mediators by peripheral immune cells.

Another mechanism by which MCP-1 may influence peripheral inflammatory responses is by affecting the production of immune regulating glucocorticoids. Glucocorticoids like corticosterone are produced by the adrenal gland after activation of the HPA axis and exert powerful anti-inflammatory properties [4]. Glucocorticoids can both suppress production of inflammatory mediators such as IL-1β, TNF-α, prostaglandins, free oxygen radicals, and nitric oxide, and also upregulate expression of anti-inflammatory cytokines such as IL-10 and TGF-β [3,33]. Further, adrenalectomized mice show increased mortality and increased plasma levels of IL-1β and TNF-α after LPS treatment and this mortality can be reversed by treatment with glucocorticoids [4]. The lower levels of corticosterone we detected in the serum of LPS-injected MCP-1^-/- ^mice may contribute to the increased levels of serum cytokines seen in these mice, but this idea needs to be further tested.

In addition to its effects on peripheral inflammatory responses, LPS administration can lead to upregulation of detrimental neuroinflammatory responses. Recent studies have shown that a single injection of a high dose of LPS (5 mg/kg) causes a decrease in neuronal numbers in the substantia nigra 10 months after injection and is correlated to increased microglial activation and TNF-α production [34]. Other studies have found long-term losses in neurons, cognitive deficits, or decreases in hippocampal size after peripheral LPS exposure [35,36]. Although a number of previous studies have examined the relationship between MCP-1 and inflammatory cytokine production in the peripheral immune system, less is known about the role of MCP-1 in CNS inflammation after LPS administration. Our data showing that a peripheral injection of LPS induces an increase in brain MCP-1 levels are consistent with a previous report showing an increase in brain MCP-1 protein levels at 1 hour after a 3 mg/kg i.p. LPS injection [37]. In other studies, MCP-1 mRNA was found to be rapidly expressed starting at 30 minutes in areas lacking the BBB and along blood vessels [14], and MCP-1 mRNA expression appeared throughout the brain parenchyma starting by 3 hours and peaking at 6–8 hours after systemic LPS administration [13]. Further, in the brain both immune cells like macrophages/microglia [14] and non-immune cells like endothelia [14] and astrocytes [13] can produce MCP-1 after peripheral LPS. These data suggest a pattern of LPS-induced MCP-1 expression that begins in the periphery and spreads to the brain, first in areas that are in contact with peripherally circulating inflammatory mediators and then throughout other areas of the brain. Other inflammatory mediators also follow a similar site-specific pattern of expression after peripheral LPS, including cyclooxygenase-2, TNF-α, IL-1β, inducible nitric oxide synthase and Iκ Bα, where these molecules are first upregulated in the choroid plexus, circumventricular organs, and/or near blood vessels [7,38-41].

Whether LPS-induced increases in MCP-1 in brain are mechanistically coupled to changes in brain inflammation has not been explored in detail. The results reported here showing that MCP-1^-/- ^mice have decreased brain inflammation after systemic LPS administration compared to wild-type mice suggest that MCP-1 has an important role in activating the brain during peripheral endotoxemia. There are several possible mechanisms by which MCP-1 can regulate neuroinflammation and the production of inflammatory mediators in the CNS.

First, MCP-1 in the brain may act as a chemoattractant to recruit microglia or other immune cells to areas of pathology or inflammation; once activated near the site of pathology, the recruited cells can produce more pro-inflammatory mediators thus increasing inflammation. This possibility is supported by observations that MCP-1 is chemotactic to microglia *in vitro *[42], and that injection of recombinant MCP-1 into the striatum or hippocampus of mice results in a significant increase in numbers of microglia/monocytes near the injection site compared to vehicle-injected mice [43,44]. Other animal models also support a chemoattractant role for MCP-1. For example, in rodent stroke models, inhibition of MCP-1 using knockout mice or anti-MCP-1 gene therapy results in fewer activated microglia and astrocytes and significantly smaller infarcts [22,45], while overexpression of MCP-1 is associated with increased chemoattraction of immune cells and larger infarct volumes [46]. MCP-1 has also been implicated in immune cell recruitment in several other CNS disorders such as demyelinating diseases, lesion models, and cortical injury [23,47,48]. Our findings of decreased activation of microglia as measured by CD45 staining in the LPS-treated MCP-1^-/- ^mice that correlates with a decrease in brain inflammatory cytokines suggest that MCP-1 is necessary for recruitment and activation of microglia. Future studies are necessary to further delineate the detailed mechanisms, such as flow cytometry to quantify cell infiltration into the brain and MCP-1 production.

Second, MCP-1 may be required for glia to mount an effective neuroinflammatory response, either through altering the balance between anti-inflammatory and pro-inflammatory responses towards a more pro-inflammatory profile or in "priming" glial cells toward a more inflammatory profile in the brain. Our results so far with the MCP-1^-/- ^mice do not indicate a major increase in anti-inflammatory mediators in the brain. For example, IL-10 levels were not different in the LPS-injected MCP-1^-/- ^mice versus the LPS-injected MCP-1^+/+ ^mice. The idea of a MCP-1 "priming" mechanism driving an inflammatory profile comes from studies showing that when LPS is injected directly into the striatum of MCP-1^-/- ^mice, there is a decrease in inflammatory cytokine production that is not correlated with activation or recruitment of microglia or brain immune cells [43]. The authors propose that low levels of MCP-1 along with other inflammatory mediators in the brain result in "priming" of the glial cells to become more responsive to later inflammatory insults, and the total inhibition of MCP-1 in the knockout model renders the glia less responsive [43]. We are currently investigating this possibility with glial cell cultures.

Third, MCP-1 may also be important in regulation of the BBB, and disruption of this regulation might lead to increased permeability of the BBB and more leukocyte infiltration from the periphery. Support for this idea comes from the finding that MCP-1 can increase the BBB permeability to FITC-albumin [20] and modulate the expression of tight junction proteins in endothelial cells of the BBB [20,49]. It is possible that MCP-1 is involved in increasing BBB permeability after an LPS insult, thereby allowing infiltration of immune cells. The lack of MCP-1 in the knockout mice would then result in less brain inflammation because of fewer infiltrating immune cells. We have not directly tested this possibility, but have observed that 4 of 5 MCP-1^+/+ ^mice injected with LPS had what appeared to be leukocyte infiltration into the brain parenchyma, whereas only 1 of 4 MCP-1^-/- ^mice showed these cells. These data indicate that the lack of MCP-1 may confer a protective effect against alterations in BBB permeability after LPS treatment.

## Conclusion

In summary, we find that MCP-1^-/- ^mice have decreased brain inflammation after a peripheral LPS insult, despite an exaggerated peripheral response. These data suggest an important role for MCP-1 in regulation of brain inflammation after peripheral endotoxemia. Although the mechanisms of activating the brain after acute peripheral inflammation are incompletely understood, our data suggest that MCP-1 may be critical in transferring inflammatory signals from the periphery to the brain.

## Abbreviations

IL: interleukin; TNF-α: tumor necrosis factor-α; MCP: monocyte chemoattractant protein; CNS: central nervous system; LPS: lipopolysaccharide; HPA: hypothalamic-pituitary-adrenal; BBB: blood brain barrier; i.p: intraperitoneal; TGF-β: transforming growth factor-β; MIP-1: monocyte inflammatory protein-1; ACTH: adrenocorticotropic hormone; M-CSF: macrophage colony stimulating factor.

## Competing interests

The authors declare that they have no competing interests.

## Authors' contributions

WT helped conceive the study, carried out the *in vivo *experiments and assays, and drafted the manuscript with the assistance of the other authors. WK provided the knockout mice and gave helpful advice and suggestions about the study. LVE helped conceive and coordinate the study, analyze data, and assist in the preparation of the manuscript. All authors read and approved the final manuscript.
